# Supplementary material for: A Comprehensive Assessment of Ultraviolet-Radiation-Induced Mutations in Flammulina filiformis Using Whole-Genome Resequencing
Source: J Fungi (Basel). 2024 Mar 20;10(3):228. doi: 10.3390/jof10030228 (PMC10971301; doi:10.3390/jof10030228)
Supplement: Supplementary file 1 [file jof-10-00228-s001.zip › Supplementary Material S8/KEGG annotation/out/64381550635650.os/KO/out_map/map05034.html]

KEGG PATHWAY: Alcoholism - Reference pathway


|  |  |
| --- | --- |
| **Alcoholism - Reference pathway** |  |

[
Pathway menu
| Organism menu
| Pathway entry
| Show description
| User data mapping
]

Alcoholism, also called dependence on alcohol (ethanol), is a chronic relapsing disorder that is progressive and has serious detrimental health outcomes. As one of the primary mediators of the rewarding effects of alcohol, dopaminergic ventral tegmental area (VTA) projections to the nucleus accumbens (NAc) have been identified. Acute exposure to alcohol stimulates dopamine release into the NAc, which activates D1 receptors, stimulating PKA signaling and subsequent CREB-mediated gene expression, whereas chronic alcohol exposure leads to an adaptive downregulation of this pathway, in particular of CREB function. The decreased CREB function in the NAc may promote the intake of drugs of abuse to achieve an increase in reward and thus may be involved in the regulation of positive affective states of addiction. PKA signaling also affects NMDA receptor activity and may play an important role in neuroadaptation in response to chronic alcohol exposure.

|  |  |
| --- | --- |
| Reference pathway | 100% |
